# Downregulation of Leucine-Rich Repeat-Containing 8A Limits Proliferation and Increases Sensitivity of Glioblastoma to Temozolomide and Carmustine

**DOI:** 10.3389/fonc.2018.00142

**Published:** 2018-05-07

**Authors:** Sebastian Rubino, Martin D. Bach, Alexandra L. Schober, Ian H. Lambert, Alexander A. Mongin

**Affiliations:** ^1^Department of Neurosurgery, Albany Medical Center, Albany, NY, United States; ^2^Department of Neuroscience and Experimental Therapeutics, Albany Medical College, Albany, NY, United States; ^3^Section for Cell Biology and Physiology, Department of Biology, University of Copenhagen, Copenhagen, Denmark

**Keywords:** glioblastoma, leucine-rich repeat-containing 8A, volume-regulated anion channel, temozolomide, carmustine, cisplatin

## Abstract

**Background:**

Glioblastoma (GBM) is the most common primary malignant brain tumor in adults. Ubiquitously expressed volume-regulated anion channels (VRAC) are thought to play a role in cell proliferation, migration, and apoptosis. VRAC are heteromeric channel complexes assembled from proteins belonging to the leucine-rich repeat-containing 8A (LRRC8A through E), among which LRRC8A plays an indispensable role. In the present work, we used an RNAi approach to test potential significance of VRAC and LRRC8A in GBM survival and sensitivity to chemotherapeutic agents.

**Methods:**

Primary GBM cells were derived from a human surgical tissue sample. *LRRC8A* expression was determined with quantitative RT-PCR and downregulated using siRNA. The effects of LRRC8A knockdown on GBM cell viability, proliferation, and sensitivity to chemotherapeutic agents were determined using 3-(4,5-dimethylthiazol-2-yl)-2,5-diphenyl tetrazolium bromide and Coulter counter assays. Cell cycle progression was further explored using fluorescence-activated cell sorting analysis of propidium iodide-stained cells.

**Results:**

Temozolomide (TMZ), carmustine, and cisplatin reduced GBM cell survival with the IC_50_ values of ~1,250, 320, and 30 µM, respectively. Two of three tested gene-specific siRNA constructs, siLRRC8A_3 and siLRRC8A_6, downregulated *LRRC8A* expression by >80% and significantly reduced GBM cell numbers. The most potent siLRRC8A_3 itself reduced viable cell numbers by ≥50%, and significantly increased toxicity of the sub-IC_50_ concentrations of TMZ (570 µM) and carmustine (167 µM). In contrast, the effects of siLRRC8A_3 and cisplatin (32 µM) were not additive, most likely because cisplatin uptake is VRAC-dependent. The results obtained in primary GBM cells were qualitatively recapitulated in U251 human GBM cell line.

**Conclusion:**

Downregulation of LRRC8A expression reduces GBM cell proliferation and increases sensitivity to the clinically used TMZ and carmustine. These findings indicate that VRAC represents a potential target for the treatment of GBM, alone or in combination with the current standard-of-care.

## Introduction

Glioblastoma (GBM) is the most common malignant primary brain tumor in adults (1, 2), with an annual incidence of approximately 4 per 100,000 people and 14,000 new diagnoses per year (3). Despite extensive translational work and clinical studies, the prognosis for GBM patients remains grim. Newly diagnosed patients typically receive maximal safe surgical resection, followed by treatment with the chemotherapeutic temozolomide (TMZ) and radiation therapy (2). However, even after receiving this standard-of-care treatment, median relative survival time ranges from 13 to 15 months only, with a 2-year overall survival of 21–26% (3, 4).

Temozolomide is the most commonly used chemotherapeutic in the treatment of GBM. It is an imidazotetrazine compound that is metabolically converted to the reactive methyldiazonium cation, which in turn methylates DNA and triggers apoptotic cell death (5, 6). GBM tumor cells develop chemoresistance by upregulating methylguanine methyltransferase to remove the cytotoxic O6-methylguanine, or *via* selection of mismatch repair-deficient cells, which can tolerate alkylating agents (5). Additional adjuvant therapy involves the surgical implantation of Gliadel wafers, which provide local delivery of the chemotherapeutic polymer carmustine (bis-chloroethylnitrosourea) (7, 8). Gliadel wafers are used less frequently because systematic review of clinical data indicates that they only increase survival marginally and are associated with high-complication rates (9). Limited efficacy of existing therapies creates an urgent need for development of novel treatment modalities.

Ion channels are frequently discussed as possible targets for cancer treatment due to their many roles in tumor biology [reviewed in Ref. (10–12)]. In the present study, we focused on the ubiquitously expressed volume-regulated chloride/anion channels (VRAC). VRAC were first functionally discovered in epithelial and immune cells (13, 14), and soon thereafter detected in numerous other cell types, including rat glioma cells (15–19). Although the primary function of VRAC is cell volume regulation, these channels are believed to play a role in cell proliferation, migration, and apoptosis, in normal and malignant cells [reviewed in Ref. (17, 19–21)]. Due to their purported significance in proliferation and migration, VRAC have long been considered a potential therapeutic target. However, the direct evidence for their contribution to these processes has been lacking due to limited specificity of pharmacological VRAC blockers, and because the molecular identity of VRAC remained elusive for nearly three decades (19).

In 2014, two groups independently identified proteins of the leucine-rich repeat-containing 8A (LRRC8A) as components of the heteromeric VRAC (22, 23). LRRC8A is mandatory for VRAC activity but this subunit has to be heteromerized with at least one additional protein from the same LRRC8 family to produce a functional, presumably hexameric channel complex (23). Our laboratory was the first to establish the indispensable role of LRRC8A in forming VRAC in rat astrocytes (24), and we subsequently found that in astroglial cells there are at least two functionally distinct LRRC8A-containing VRAC heteromers (25). As GBM tumors are thought to originate from anaplastic astroglia or glial progenitor cells (26, 27), the prior work prompted us to test if the LRRC8-containing channels are important for GBM cell proliferation. Furthermore, we explored whether targeting VRAC could interfere with the effect of the clinically used chemotherapeutic agents, TMZ, and carmustine. Given the prior findings that the LRRC8A/LRRC8D-containing VRAC facilitate cisplatin sensitivity in several cell types (28, 29), we used cisplatin as a reference compound.

## Materials and Methods

### Materials and Reagents

Temozolomide, carmustine, and 3-(4,5-dimethylthiazol-2-yl)-2,5-diphenyl tetrazolium bromide (MTT) were purchased from Millipore-Sigma (St. Louis, MO, USA). Cisplatin was acquired from Tocris/Bio-Techne (Minneapolis, MN, USA). Lipofectamine RNAiMax, DNase-free RNase A, and 1 mg/ml stock solution of propidium iodide in water were from Thermo Fisher Scientific (Waltham, MA, USA). Cell culture components—fetal bovine serum (FBS), glutamine-containing Earl’s minimal essential medium (MEM, cat. # 10,293), OptiMEM, penicillin plus streptomycin, and the recombinant protease TrypLE Express, all of the Gibco brand—were from Thermo Fisher Scientific. Quantitative PCR primers for *LRRC8A* and the housekeeping genes *RPL13a, RPS20*, and *GAPDH*, gene-specific siRNAs and negative control Allstars siRNA were all purchased from Qiagen (Germantown, MD, USA). All other salts and reagents were purchased from Millipore-Sigma, and were of analytical grade, unless specified otherwise.

Carmustine and TMZ were dissolved under sterile conditions in dimethyl sulfoxide (DMSO) to stock concentrations of 30 and 150 mM, respectively, and stored at −20°C until used in experiments. Cisplatin was dissolved in sterile Dulbecco’s phosphate-buffered saline (DPBS) to produce 3.3 mM stock solution, and stored at −20°C until used.

### Primary Culture of GBM and U251 MG Cell Line

Primary GBM cells, which had been characterized in our prior work, were prepared from a surgical GBM tissue sample as previously described (30). The specimen was obtained under the protocol approved by the Albany Medical Center Institutional Review Board, with written patient consent. The GBM origin of tissue was histologically confirmed by a pathologist at the time of resection. Briefly, tumor tissue was washed with ice-cold Ca^2+^/Mg^2+^-free phosphate-buffered saline (PBS, pH 7.4), dissected into small pieces, digested in the solution of 0.125% trypsin/0.015% EDTA, which additionally contained DNAse I, and further gently dissociated using trituration with a fire-polished glass Pasteur pipette. The dissociated cells were initially propagated in T75 cell culture flasks in DMEM plus 20% FBS supplemented with 100 U/ml penicillin, and 100 µg/ml streptomycin. After two passages, the FBS content was reduced to 10%, and penicillin and streptomycin concentrations were reduced to 50 U/ml and 50 µg/ml, respectively. Propagated cells were removed from the substrate using recombinant protease TrypLE, transferred into freezing medium, and stored in liquid nitrogen.

For the presented work, GBM cells were re-plated in MEM supplemented with 10% FBS, 50 µg/ml penicillin, and 50 µM streptomycin, and grown in T75 flasks in the humidified atmosphere of 5% CO_2_/balance air at 37°C. Cells were periodically passaged after they reached 70–90% confluence, or used for experimental procedures as described below. All presented experiments were carried out with cells in passages 3 through 13.

For comparative purposes, we used human GBM cell line U251 MG (hereafter referred to as U251). This cell line is originally derived in the early 1970s from a male patient with malignant grade III–IV astrocytoma (31). U251 utilized in this study was a gift of Dr. Michael G. Kaplitt. The initial passage of these cells is unknown. Among other features, U251 express a mutated p53 protein, which is typical for GBM tumors, and they are positive for the astroglial cell marker, glial fibrillary acidic protein (GFAP) (32). Expression of human cell markers and GFAP immunoreactivity has been confirmed in our prior published work (33). U251 cells were propagated in MEM supplemented with 10% FBS and antibiotics, as described above. The cultivation and assay conditions were the same as for primary GBM cells.

### Quantitative RT-PCR (qRT-PCR) Analysis of Gene Expression

Relative expression of *LRRC8A* mRNA and the efficacy of siRNA gene knockdowns were determined using qRT-PCR. GBM cells were plated in 60-mm Petri dishes. Non-transfected or siRNA-transfected cells were grown for 48 h. mRNA species were isolated using the RNAqueous-4PCR kit (Thermo Fisher Scientific) according to the manufacturer’s protocol. mRNA was converted to cDNA using the iScript cDNA synthesis kit (Bio-Rad Laboratories, Hercules, CA, USA). Human *LRRC8A* expression levels were determined using quantitative PCR primers (Qiagen cat. #QT01023302) and SYBR Green master mix (Bio-Rad). The expression levels were normalized within each sample to the housekeeping genes, the ribosomal proteins *RPL13A* (cat.# QT00089915), and *RPS20* (cat.# QT01666847). Real-time detection of PCR products was done using a CFX96 Real-Time PCR setup (Bio-Rad).

### siRNA Transfections

Glioblastoma cell transfection was performed as previously described (24). Briefly, primary GBM or U251 cells were plated in 24-well cell culture plates or 60-mm Petri dishes (10,000 cells per well or ~150,000 cells per Petri dish) and grown for 1 (U251) or 3 days (primary GBM) prior to transfection. The cell culture medium was removed and cells were washed with the serum-free OptiMEM, before transfection in the same medium with either negative control siRNA or siRNA targeting *LRRC8A*, using the Lipofectamine RNAiMax transfecting agent (Invitrogen/Thermo Fisher Scientific, Waltham, MA, USA). We used three different gene-specific siRNA constructs: siLRRC8A_3 (Hs_LRRC8A_3; Qiagen cat.# SI04251807), siLRRC8A_4 (Hs_LRRC8A_4; cat.# SI04327001), and siLRRC8A_6 (Hs_LRRC8A_6; cat.# SI05006036), and AllStars scrambled siRNA as a negative control. siRNA-transfection reagent complexes were prepared in OptiMEM per the manufacturer’s instructions, and then further diluted with OptiMEM to the final siRNA concentration of 50 nM and added to the wells. After 4 h incubation with siRNA complexes, threefold excess of the FBS-containing cell culture medium was added on the top, and cells were further grown for ~44 h prior to adding chemotherapeutic agents. 48 h after adding chemotherapeutics (four full days of transfection) changes in cell proliferation and viability were determined using an MTT assay.

### MTT Assay

The MTT cell proliferation and viability assay was conducted as previously described (33). Briefly, GBM cells were plated into 24-well plates at either “lower” (10,000 cells/well) or “higher” (40,000 cells/well) density and allowed to grow for one (U251) or three (primary GBM) days prior to experimental treatments. Cells were subsequently treated with chemotherapeutic agents or transfected with siRNA as described above. After completing cell treatments, cell culture medium was removed by aspiration, and cells were washed once with the chemically defined basal solution containing (in millimolar): 135 NaCl, 3.5 KCl, 1.2 MgSO_4_, 1.3 CaCl_2_, 1.2 KH_2_PO_4_, 10 HEPES, and 10 D-glucose (pH 7.4, adjusted with NaOH). Subsequently, the basal medium was substituted with the same solution additionally containing 0.5 mg/ml MTT. Depending on cell density, MTT incubation lasted 1–2 h. The MTT solution was removed by inverting the plate without washing. The metabolically formed formazan crystals were dissolved using 1 ml of acidified isopropanol and the absorbance of the solution in each well was determined at 562 nm, using a BioTek ELx800 plate reader (Winooski, VT, USA).

### Coulter Counter Assay

To independently verify the MTT assay results, we directly counted GBM and U251 cells using a Coulter counter as described before (33). Briefly, cells grown in 24-well plates at plating density as above were detached from the substrate using a recombinant protease TrypLE and resuspended in the ISOTON II diluent (Beckman Coulter, Miami, FL, USA). Cell numbers were determined using a Z2 series Coulter counter (Beckman Coulter), with the detection cell diameter limits set at 7–25 µm.

### Flow Cytometry Cell Cycle Analysis

Cell cycle analysis was performed by quantitation of the relative DNA content using fluorescence-assisted cell sorting (FACS) of propidium iodide-stained cells as originally described by Krishan (34) with further extension for analysis of apoptosis (35, 36). Briefly, cells were plated and treated in 12-well plates as described for MTT assays. They were detached from the substrate with TrypLE, fixed for 20 min at room temperature (20°C) in a hypotonic solution containing 2% paraformaldehyde, and rinsed from the fixative by sedimentation in PBS. Fixed cells were stained in the PBS solution containing 1 mg/ml DNase-free RNase A and 10 µg/ml propidium iodide for 30 min at room temperature. Single cell propidium iodide fluorescence was quantified using an FACSCanto flow cytometer (Becton Dickinson Biosciences, San Jose, CA, USA) and analyzed with FlowJo 7.5 software (FlowJo, LLC., Ashland, OR, USA).

### Statistical Analysis

All data are presented as the mean values ± SEM, after normalization to control values within the same experiment. Because of normalization, all comparisons to control values were performed using one-tailed *t*-test, followed by the Bonferroni *post hoc* correction for multiple comparisons. When applicable, comparisons between experimental groups were done using two-tailed *t*-test with Bonferroni *post hoc* correction. Origin 8.1 software (OriginLab, Northampton, MA, USA) was used for statistical analyses. A *p*-value of <0.05 was considered statistically significant.

## Results

### *LRRC8A* Gene Expression and Validation of siRNA Tools

**Figure 1:**
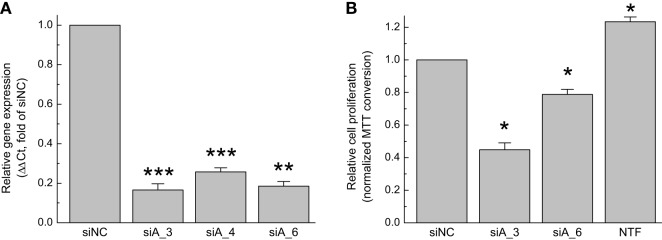
Downregulation of leucine-rich repeat-containing 8A (LRRC8A) protein limits proliferation of primary glioblastoma (GBM) cells. **(A)** Validation of siRNA constructs. GBM cells were transfected with three gene-specific siRNA constructs, siLRRC8A_3 (siA_3), siLRRC8A_4 (siA_4), or siLRRC8A_6 (siA_6). Changes in the LRRC8A mRNA expression levels were quantified 48 h post-transfection and normalized to the expression levels of the housekeeping gene RPL13a in the same samples. The data are the mean values ± SE of four independent experiments performed in triplicates (*n* = 4). **p < 0.01, ***p < 0.001 vs. cells transfected with negative control siRNA (siNC). **(B)** Effect of gene-specific LRRC8A siRNA on proliferation of GBM cells. Cells were transfected with LRRC8A siRNA (siA_3 or siA_6), or siNC. Relative proliferation rates were determined 96 h post-transfection using a 3-(4,5-dimethylthiazol-2-yl)-2,5-diphenyl tetrazolium bromide (MTT) assay, and further normalized to siNC values within the same experiment. Non-transfected cells (NTF) were used as an additional internal control. The data are the mean values ± SE of three independent experiments performed in triplicates (*n* = 3). *p < 0.05 vs. cells transfected siNC.

In order to test functional significance of the LRRC8A-containing VRAC, we initially verified its expression in GBM cells and validated *LRRC8A*-targeting siRNA constructs using quantitative real-time reverse transcriptase PCR (qRT-PCR) approach. In the GBM cell line used in the present study, *LRRC8A* mRNA expression was 0.27 ± 0.17% when normalized to the housekeeping gene *RPL13A* within the same cDNA samples (*n* = 5 independent cell preparations). These values are comparable with those which we found in primary astroglial cells isolated from the rodent brain (25). We next tested three different siRNA constructs for their ability to knockdown *LRRC8A* expression. In order of potency, siLRRC8A_3, siLRRC8A_6, and siLRRC8A_4 reduced the *LRRC8A* mRNA levels by 84% (*p* < 0.001), 82% (*p* < 0.01), and 73% (*p* < 0.001), respectively (Figure 1A). We further tested siLRRC8A_3 and siLRRC8A_6 for their effects on cell proliferation and found that siLRRC8A_3 and siLRRC8A_6 decreased GBM proliferation by 55% (*p* < 0.05) and 21% (*p* < 0.05), respectively (Figure 1B). The most efficacious siLRRC8A_3 was used in all subsequent cell viability assays.

### Characterization of GBM Sensitivity to Chemotherapeutic Agents

Glioblastoma cells are known to have variable sensitivity to chemotherapeutic agents and to develop chemoresistance (37–40). As we used a primary GBM cell line, we first performed a dose–response study to determine IC_50_ values for TMZ, carmustine, and cisplatin. We included cisplatin as an experimental control, because it enters cancer cells *via* VRAC (28, 29).

**Figure 2:**
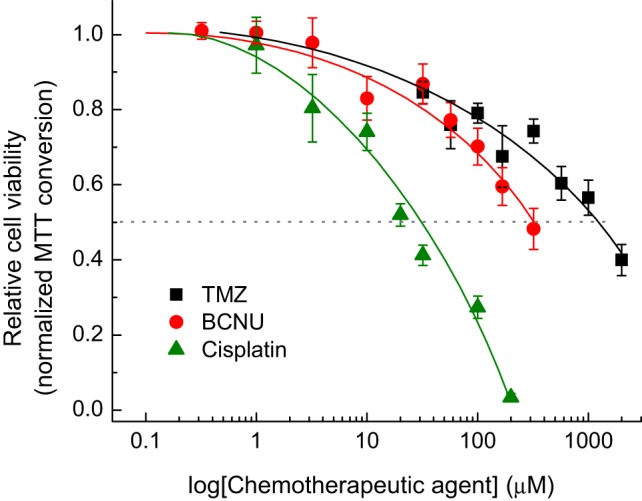
Dose-dependent effects of the chemotherapeutic agents temozolomide (TMZ), carmustine [bis-chloroethylnitrosourea (BCNU)], and cisplatin on viability of primary glioblastoma (GBM) cells. GBM cells were treated with various concentrations of chemotoxic agents and the relative cell numbers were compared 48 h later using a 3-(4,5-dimethylthiazol-2-yl)-2,5-diphenyl tetrazolium bromide (MTT) assay. Each experimental point represents the mean values ± SE of four to eight independent measurements, which were accumulated in four independent experiments and normalized to an average MTT signal in non-treated cells within the same experiment. The three lowest concentrations of BCNU were tested two times.

As shown in Figure 2, all three chemotherapeutic agents reduced viability of GBM cells in a dose-dependent manner. Cisplatin showed the highest toxicity, whereas TMZ was least toxic. The IC_50_ values for cisplatin, carmustine, and TMZ, were approximately 30, 320, and 1,250 µM, respectively (Figure 2). Due to TMZ and carmustine’s hydrophobicity, their stock solutions were prepared in DMSO before addition to experimental media. However, even in DMSO, TMZ and carmustine have a low solubility. Consequently, the three highest tested dosages of these agents were delivered with DMSO levels between 0.5 and 1.5%. To control for vehicle toxicity, we tested the effect of DMSO on cell viability. DMSO alone reduced cell numbers, in a dose-dependent manner (Figure S1 in Supplementary Material). These findings are consistent with the prior reports that in some cell types proliferation can be inhibited by DMSO levels as low as 0.1% (41, 42). To reduce DMSO toxicity, further individual proliferation experiments were carried out at the sub-IC_50_ levels of TMZ (570 µM) and carmustine (167 µM) and 0.38% DMSO was used as a vehicle control. Cisplatin was dissolved in DPBS, and further tested at the IC_50_ concentration (32 µM). No vehicle control was necessary in the latter case.

### *LRRC8A* Knockdown Decreases GBM Proliferation and Increases Sensitivity to TMZ and Carmustine, but Not Cisplatin

**Figure 3:**
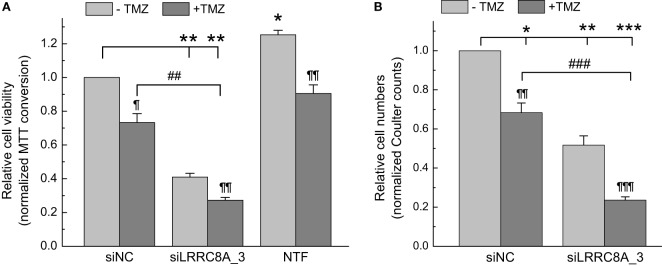
Effects of temozolomide (TMZ) and leucine-rich repeat-containing 8A (LRRC8A) siRNA on viability and proliferation of glioblastoma (GBM) cells determined by 3-(4,5-dimethylthiazol-2-yl)-2,5-diphenyl tetrazolium bromide (MTT) **(A)** or Coulter counter **(B)** assays. GBM cells were transfected with siLRRC8A_3 or negative control siRNA (siNC). 48 h post-transfection cells were additionally treated with 570 µM TMZ. Relative levels of cell proliferation were determined 48 h after addition of TMZ (96 h post-transfection with siRNA). Non-transfected cells (NTF) were used in MTT assays as an additional internal control. The data are the mean values ± SE of three independent experiments performed in triplicates (*n* = 3) for MTT assays, or four independent transfections (*n* = 4) in Coulter counter experiments. *p < 0.05, **p < 0.01, ***p < 0.001 vs. cells transfected with siNC. ^¶^*p* < 0.05, ^¶¶^*p* < 0.01, ^¶¶¶^*p* < 0.001, within the group effect of TMZ vs. siRNA or mock treatment alone. ^##^*p* < 0.01, ^###^*p* < 0.001, TMZ effect in cells treated with siNC vs. cells treated with siLRRC8A_3.

**Figure 4:**
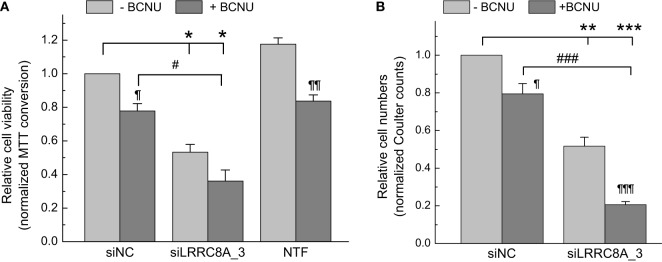
Effects of carmustine [bis-chloroethylnitrosourea (BCNU)] and leucine-rich repeat-containing 8A (LRRC8A) siRNA viability and proliferation of glioblastoma (GBM) cells determined using 3-(4,5-dimethylthiazol-2-yl)-2,5-diphenyl tetrazolium bromide (MTT) **(A)** or Coulter counter **(B)** assays. GBM cells were transfected with siLRRC8A_3 (siA_3) or negative control siRNA (siNC). 48 h post-transfection cells were additionally treated with 167 µM BCNU. Relative levels of cell proliferation were determined 48 h after addition of BCNU (96 h post-transfection with siRNA) using an MTT assay. Non-transfected cells (NTF) were used as an additional internal control in MTT experiments. The data are the mean values ± SE of three independent experiments performed in triplicates (*n* = 3) in MTT assays, or four independent transfections (*n* = 4) in Coulter counter experiments. *p < 0.05, **p < 0.01, ***p < 0.001 vs. cells transfected with siNC. ^¶^*p* < 0.05, ^¶¶^*p* < 0.01^, ¶¶¶^*p* < 0.001, within the group effect of BCNU vs. siRNA or mock treatment alone. ^#^*p* < 0.05, ^###^*p* < 0.001, BCNU effect in cells treated with siNC vs. cells treated with siLRRC8A_3.

**Figure 5:**
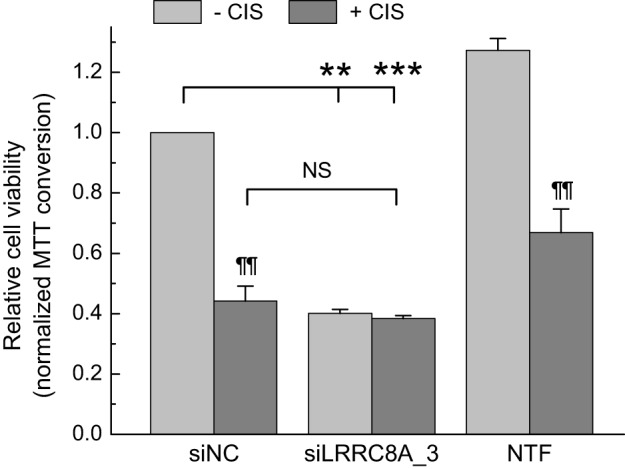
Effects of cisplatin and leucine-rich repeat-containing 8A (LRRC8A) siRNA on viability of glioblastoma (GBM) cells. GBM cells were transfected with siLRRC8A_3 or negative control siRNA (siNC). 48 h post-transfection cells were additionally treated with 30 µM cisplatin or matching concentration of the vehicle (Dulbecco’s phosphate-buffered saline). Cell viability was determined 48 h after addition of cisplatin (96 h post-transfection with siRNA) using an 3-(4,5-dimethylthiazol-2-yl)-2,5-diphenyl tetrazolium bromide (MTT) assay. Non-transfected cells (NTF) were used as an additional internal control. The data are the mean values ± SE of three independent experiments performed in triplicates (*n* = 3). **p < 0.01, ***p < 0.001 vs. cells transfected with siNC. ^¶¶^*p* < 0.01, within the group effect of cisplatin vs. siRNA or mock treatment alone. NS, not significant, cisplatin effects in cells treated with siNC vs. cells treated with siLRRC8A_3.

As illustrated in Figures 3–5, knockdown of *LRRC8A* consistently decreased GBM cell numbers by ~50%, pointing to the likely importance of VRAC in GBM cell proliferation. To explore if *LRRC8A* knockdown impacts sensitivity of GBM cells to clinically used chemotherapeutic agents, we tested the effects of TMZ and carmustine in conjunction with the *LRRC8A*-targeting siRNA. As seen in Figure 3A, downregulation of *LRRC8A* strongly and significantly reduced MTT signal by 59% (*p* < 0.01), while TMZ was less potent (27% reduction, *p* < 0.05). The effect of combining *LRRC8A* siRNA with TMZ approached the level of additivity of individual treatments (73%, *p* < 0.01, Figure 3A). Although the MTT assay fairly faithfully reflects relative cell numbers (43), we and others have encountered situations where formazan production was reduced without changes in cell viability [see, for example, (44)]. In order to validate MTT results, we repeated cell treatments and directly counted cells using a Coulter counter. As shown in Figure 3B, the results of electronic cell counts were quantitatively very similar: 49, 21, and 79% for LRRC8A siRNA (*p* < 0.01), TMZ (*p* < 0.05), and their combination (*p* < 0.001), respectively. These findings reaffirm the conclusions on reduced cell proliferation after LRRC8A knockdown, and additivity of siRNA and TMZ effects.

In the next series of experiments, we observed very similar phenomena in GBM cells treated with another clinically utilized chemotherapeutic, carmustine. Specifically, siRNA, carmustine, and their combination decreased cell viability by 47% (*p* < 0.05), 22% (*p* < 0.05), and 64% (*p* < 0.05), respectively (Figure 4A). In the latter case, due to experimental variability there was only a trend for additive actions of carmustine with siRNA transfection (*p* < 0.1). In the Coulter counter experiments shown in Figure 4B, the same treatments reduced cell numbers in a nearly identical fashion: 49% (*p* < 0.01), 21% (*p* < 0.05), and 79% (*p* < 0.001), for siRNA, carmustine, and their combination, respectively. In these latter assays, the additive toxicity of the LRRC8A knockdown and carmustine was very clear and statistically significant.

We additionally tested the chemotherapeutic agent cisplatin. This was important because prior work in the field established that the LRRC8A/LRRC8D-containing VRAC is largely responsible for cisplatin uptake (28, 29). In contrast to TMZ and carmustine, downregulation of *LRRC8A* eliminated sensitivity of GBM cells to cisplatin. The individual treatments of siRNA or cisplatin reduced GBM cell viability by 60% (*p* < 0.01) and 56% (*p* < 0.01), respectively (Figure 5). Combination of siRNA plus cisplatin did not produce significant additional effect, reducing cell viability by 62% (Figure 5, *p* = 0.38, compared with siRNA alone; *p* = 0.32, compared with cisplatin alone).

### Cell Cycle Analysis in Cells Treated With the LRRC8A siRNA and Chemotherapeutics

**Figure 6:**
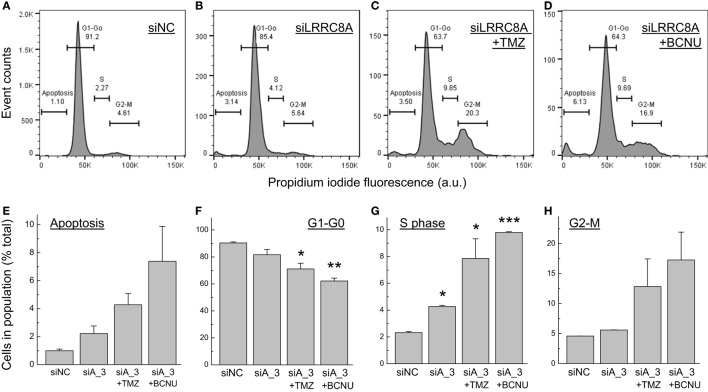
Fluorescence-assisted cell sorting (FACS) analysis of cell proliferation in glioblastoma (GBM) cells treated with leucine-rich repeat-containing 8A (LRRC8A) siRNA and chemotherapeutics agents. DNA content in fixed and permeabilized GBM cells was determined after staining with propidium iodide using FACS analysis, as described in Section “Materials and Methods.” **(A–D)** Representative FACS data in cells treated with negative control siRNA (siNC), LRRC8A siRNA (siA_3), or their combination, as specified. **(E–H)** Quantitation of FACS data for cells segregated in apoptotic **(E)**, G_1_–G_0_ **(F)**, S phase **(G)**, and G_2_/M **(H)** populations. The data are the mean values ± SE of three independently transfected cell populations. *p < 0.05, **p < 0.01, ***p < 0.001, vs. cells transfected with siNC.

In order to gain insight into potential mechanisms responsible for the impact of LRRC8A downregulation on cell viability and proliferation, we performed FACS analysis of propidium iodide-stained cells (34–36). The results of these experiments are presented in Figure 6. LRRC8A knockdown produced statistically significant increases in cells undergoing the S phase of mitosis (~twofold increases, *p* < 0.05, Figure 6G). We also found a not-significant trend for increases in the apoptotic cell numbers (Figure 6E). These changes were associated with matching decreases in the G_1_/G_0_ cell population (Figure 6F). When cells were treated with a combination of siRNA and chemotherapeutic agents, the resulting changes were much more profound. Both TMZ and carmustine produced three- to fourfold increases in the G_2_/M population (Figure 6F), similar three- to fourfold increases in S phase cells (Figure 6G), and a not significant trend of four- to sevenfold increases in apoptotic cell numbers (Figure 6E).

### Effect of the *LRRC8A* Knockdown on Viability and Cell Cycle Distribution of U251 Cells

**Figure 7:**
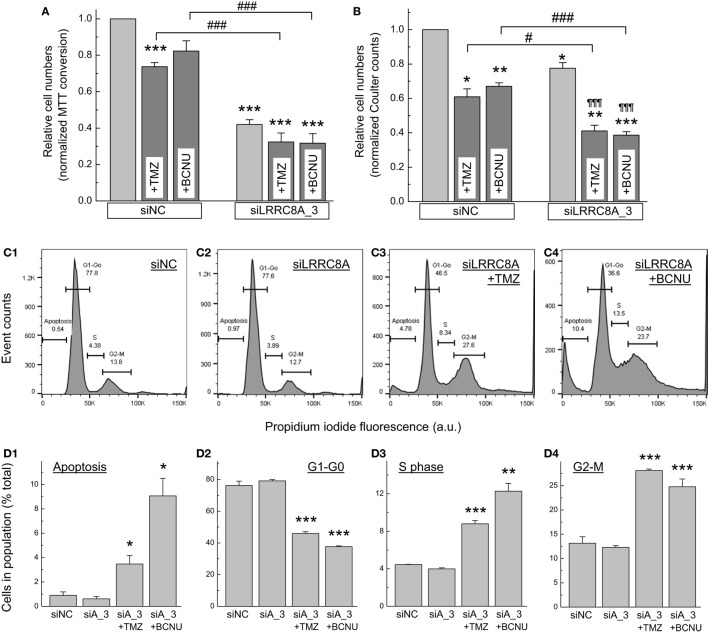
Effects of temozolomide (TMZ), carmustine [bis-chloroethylnitrosourea (BCNU)], and leucine-rich repeat-containing 8A (LRRC8A) siRNA on viability and proliferation of U251 cells determined using 3-(4,5-dimethylthiazol-2-yl)-2,5-diphenyl tetrazolium bromide (MTT) **(A)** and Coulter counter **(B)** assays, and fluorescence-assisted cell sorting (FACS) analysis **(C,D)**. U251 cells were transfected with siLRRC8A_3 (siLRRC8A or siA_3) or negative control siRNA (siNC). 48 h post-transfection they were additionally treated with 570 µM TMZ or 57 µM BCNU. Viability assays were performed 48 h after addition of chemotherapeutic agents (96 h post-transfection with siRNA). Representative FACS data are shown in the fields **C1–C4**, and their quantitation in **D1–D4**. The data are the mean values ± SE of (a) 10–12 independent MTT assays in three independently transfected U251 cell cultures, (b) four Coulter counter assays, and (c) three FACS analyses performed in independently transfected U251 samples. *p < 0.05, **p < 0.01, ***p < 0.001, vs. cells transfected with siNC. ^¶¶¶^*p* < 0.001, within the group effect of chemotherapeutics. ^#^*p* < 0.05, ^###^*p* < 0.001, the chemotherapeutics effects in cells treated with siNC vs. cells treated with siLRRC8A_3.

For comparative purposes, we recapitulated cell viability experiments in the U251 GBM cell line. We treated U251 cells with the *LRRC8A*-targeting siRNA with or without the chemotherapeutic agents, 570 µM TMZ or 57 µM carmustine. As a control, cells were treated in the same fashion but with scrambled siRNA. As seen in Figure 7A, under “control” conditions (negative control siRNA), TMZ reduced cell viability by ~25% (*p* < 0.001), while carmustine was less effective (trend for 12% inhibition, *p* = 0.055, after Bonferroni correction). In contrast, treatment with LRRC8A siRNA potently reduced cell numbers by ~55% (*p* < 0.001). When the LRRC8A siRNA was combined with TMZ or carmustine, increase in inhibition of cell viability was not statistically significant (*p* = 0.118 and 0.108, respectively, after Bonferroni correction, but see significant additivity in Figure 7B). When cell viability experiments were replicated using Coulter counter assays, the results were qualitatively similar (Figure 7B). The only difference was the weaker reductions in cell viability by the LRRC8A siRNA, perhaps reflecting less effective knockdown of the LRRC8A protein.

Fluorescence-assisted cell sorting cell cycle assays in U251 cells showed the patterns of distribution of among the three mitotic phases and apoptotic population, which were similar to the results in primary GBM. However, we found a number of quantitative and qualitative differences. In the control treatment group, we found more cells moving through S and G_2_/M phases, which likely reflects the higher proliferative potential in this cell line, when compared to primary GBM. An additional difference was the presence of polyploid cells, which had been reported in the previous studies [see, for example, (45)]. Although we identified no obvious quantitative difference between control and the *LRRC8A* siRNA-treated cells, combination of siRNA with chemotherapeutic agents strongly increased the S phase and G_2_/M phase populations, as well as enrichment of apoptotic cells (see representative graphs in Figures 7C1–4 and quantitation in Figures 7D1–4). There were also increases in polyploid cell numbers.

## Discussion

The present work was instigated to explore the functional significance of the LRRC8A-containing VRAC in human gliomas. Two major findings of our work are: (i) downregulation of LRRC8A strongly decreases numbers of GBM cells, suggesting the importance of the LRRC8A-containing VRAC for their proliferation and viability; (ii) downregulation of LRRC8A increases sensitivity of primary GBM cells to the clinically used chemotherapeutic agents TMZ and carmustine, indicating that VRAC can be targeted for therapeutic purposes.

Volume-regulated anion channels activity is firmly associated with the ability of cells to regulate their volume, and is reported to play a role in cell proliferation, migration, apoptosis, cell excitability, and transepithelial ion transport (17, 19, 21, 46). However, the collective evidence for these functional roles was rather weak due to the unknown molecular identity of VRAC and limited selectivity of available pharmacological tools. In 2014, two breakthrough publications independently identified VRAC as heteromeric channels assembled from the proteins belonging to the LRRC8 family (22, 23). Prior to this discovery, the idea of VRAC involvement in cell proliferation was supported by two types of observations: (i) non-specific VRAC inhibitors were found to strongly limit cell proliferation in numerous cell types, including malignant cells, and (ii) in dividing cells, the density of VRAC currents fluctuated depending on the cell cycle stage (47–53). We believe that the present work is the first study to use a gene-specific RNAi approach to validate the significance of LRRC8A in cell proliferation. The inhibitory effects of the LRRC8A knockdown were quantitatively similar in primary GBM cells and U251 GBM cell line. This was recapitulated with two different cell proliferation and viability assays, e.g., electronic cell counting and MTT assay. Changes in absolute cell numbers (Coulter counter) point to the reduced proliferation, but we cannot exclude other mechanisms (see discussion below).

The idea that VRAC activity is involved in control of cell proliferation is important in the context of cancer cell biology. The role of VRAC in proliferative control is supported by the prior studies utilizing pharmacological inhibitors. For example, VRAC blockers arrest small cell lung cancer, cervical cancer, and T-lymphoma cells in G_0_/G_1_ phase, based on the results of cell cytometry and changes in the expression levels of cyclins and CDK kinases (50, 53). In astroglial cells, which share cell lineage with GBM, the VRAC blocker DCPIB also causes G_0_/G_1_ arrest due to cell failure to pass the G_1_/S checkpoint (54). The latter conclusion has been derived using FACS analysis, BrdU labeling, and assays of cyclin D1 and CDK4 expression (54). In our present work, knockdown of LRRC8A caused ~twofold enrichment in cells allocated in the synthesis (S) phase of mitosis, and quantitatively similar but not statistically significant increases in the apoptotic population. These data differ from prior findings in astrocytes and do not provide a clear picture of the cell cycle arrest. The discrepancies may be explained by differences in cell type or treatment (siRNA vs. channel blocker). It is also possible that our data were skewed by the loss of cell subpopulations during fixation and staining procedures. Further work is needed to verify these findings and directly compare the effects of the LRRC8A knockdowns to the actions of pharmacological blockers.

As downregulation of the LRRC8A expression strongly reduced cell GBM cell numbers, our next important question was if targeting VRAC could enhance the effect of the chemotherapeutics, TMZ and carmustine, which are currently used in clinical settings. The outcome of the combined treatment (siRNA plus chemotherapeutics) was difficult to predict based on the existing knowledge in the field. Prior to discovery of LRRC8 proteins, development of resistance to cisplatin and few other chemotherapeutic agents has been associated with dramatic downregulation of VRAC activity and decreased ability of cells to regulate their volume (55–57). Recently, the platinum-based chemotherapeutics, cisplatin, and carboplatin have been found to accumulate in cancer cells *via* the LRRC8A/LRRC8D-containing heteromers, which were downregulated with the development of chemoresistance (28, 29). An additional complication of knocking down LRRC8A in tumor cells, which is unrelated to the transport of chemotoxic substances, arises from the established role of VRAC in apoptosis. VRAC contributes to initial apoptotic cell volume decrease, and is required for progression to the later stages of apoptosis (46, 58–61). Keeping these caveats in mind, we explored if LRRC8A knockdown would lead to undesirable chemoresistance or apoptotic resistance in GBM cells. Fortunately, we did not find that to be the case. Downregulation of LRRC8A expression in primary GBM cells produced additive toxic effects with TMZ and carmustine. In FACS assays, combinatory treatment with siRNA and chemotherapeutics consistently increased cell numbers in S and G_2_/M phases of mitosis, consistent with well-known G_2_/M arrest caused by TMZ and carmustine (62, 63), but also significant increases in numbers of apoptotic cells.

To ensure that our observations in the primary GBM cell culture are broadly applicable to other types of GBM cells, we evaluated the effects of LRRC8A knockdown on the viability of the human U251 cells, one of the most widely used GBM cell lines. Much like in the primary GBM cultures, knockdown of LRRC8A reduced U251 cell numbers by >50% and was significantly more potent than treatment with either TMZ or carmustine alone. When we combined the LRRC8A siRNA with chemotherapeutics, our assays again showed a strong trend for additivity in the toxic effects of both treatments, and were far superior to the chemotherapeutics alone. Combinatory treatment facilitated G_2_/M arrest of GBM cells and was associated with an increase in apoptotic cell numbers. Therefore, U251 results reiterate our GBM data that targeting LRRC8A can be combined with existing chemotherapies.

Our understanding of GBM biology and pathophysiology continues to evolve due to advances in the fields of GBM genetics, epigenetics, and proteomics (64–66). As already mentioned, ion channels have been linked to many aspects of tumor pathophysiology and, therefore, receive increasing attention as potential biomarkers and/or therapeutic targets (10, 67, 68). GBM proliferation, migration, and invasion are dependent on expression and activity of several types of K^+^, Ca^2+^, non-selective cation, and Cl^−^ channels (11, 30, 69–71). Among Cl^−^ channels, ClC-2, -3, and -5, and chloride intracellular channels CLIC-1 and CLIC-4 have been implicated in GBM cell migration or associated with negative clinical outcomes (72, 73). Here, we add the pore-forming subunit of VRAC, LRRC8A, to the list of ion channel proteins potentially responsible for the aggressive nature of GBM. Importantly, we tested pathological relevance of the LRRC8A-containing VRAC in the primary GBM cells, in order to avoid limitations associated with using over-passaged glioma cell lines.

In the context of development of new treatment modalities, our present findings can be summarized as follows: (a) compared with TMZ and carmustine, the LRRC8A knockdown was *twice* as effective in reducing GBM cell numbers (20–25 vs. 50–60% for chemotherapeutic agents and siRNA, respectively). (b) The combinatory treatment was *three times* more effective in reducing cell numbers, when compared with TMZ or carmustine alone (the combined toxicity reached 65–80% levels). Due to the diverse nature of GBM, our work will need to be further corroborated in additional GBM cell lines and in animal GBM models. These limitations notwithstanding, the LRRC8A-containing VRAC appears to represent a new target for the treatment of GBM, alone or in combination with the current standard-of-care.

## Ethics Statement

Primary glioblastoma cells, which had been characterized in our prior work, were prepared from a surgical GBM tissue sample as previously described (30). The specimen was obtained under the protocol approved by the Albany Medical Center Institutional Review Board, with written patient consent. The GBM origin of tissue was histologically confirmed by a pathologist at the time of resection.

## Author Contributions

SR, MB, IL, and AM conceived and designed the project. SR, MB, and AS conducted the research. SR, MB, AS, and AM analyzed the data. SR, MB, AS, IL, and AM wrote or edited the manuscript. All authors agree to be collectively responsible for the work.

## Conflict of Interest Statement

The authors declare that this study was conducted in the absence of any commercial or financial relationships that could be construed as a potential conflict of interest.

## Supplementary Material

Figure S1 Effects of the vehicle agent dimethyl sulfoxide (DMSO) on proliferation of glioblastoma (GBM) cells. GBM cells were grown in the presence of indicated concentrations of DMSO, and their relative proliferation rates were determined 48 h after addition of the tested agent with an MTT assay. The data are the mean values ± SE of four independent experiments. **p*<0.05 vs. control cells grown in the absence of DMSO.

## Abbreviations

DPBS, Dulbecco’s phosphate-buffered saline; FBS, fetal bovine serum; LRRC8A, leucine-rich repeat-containing 8A; MEM, minimal essential medium; VRAC, volume-regulated anion channels.
