# Supplementary material for: Downregulation of Leucine-Rich Repeat-Containing 8A Limits Proliferation and Increases Sensitivity of Glioblastoma to Temozolomide and Carmustine
Source: Front Oncol. 2018 May 7;8:142. doi: 10.3389/fonc.2018.00142 (PMC5949383; doi:10.3389/fonc.2018.00142)
Supplement: Figure S1 — Effects of the vehicle agent dimethyl sulfoxide (DMSO) on proliferation of glioblastoma (GBM) cells. GBM cells were grown in the presence of indicated concentrations of DMSO, and their relative proliferation rates were determined 48 h after addition of the tested agent with an MTT assay. The data are the mean values ± SE of four independent experiments. *p<0.05 vs. control cells grown in the absence of DMSO. [file Image_1.PDF]

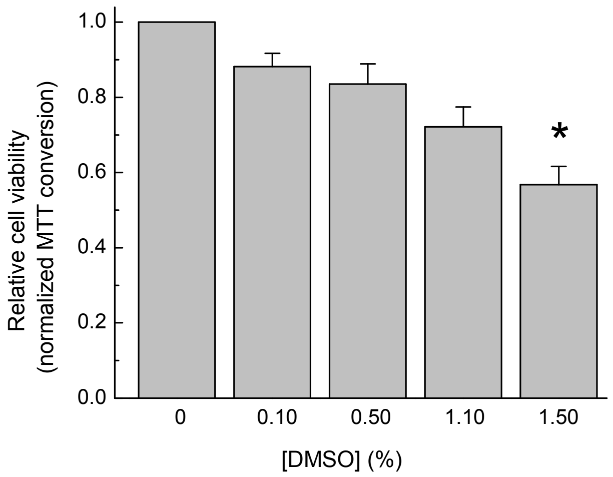

Effects of the vehicle agent DMSO on proliferation of GBM cells

GBM cells were grown in the presence of indicated concentrations of DMSO, and their relative proliferation rates were determined 48 h after addition of the tested agent with an MTT assay. The data are the mean values ±SE of four independent experiments. *$p<0.05$ vs. control cells grown in the absence of DMSO.
